# Administration of rIL-33 Restores Altered mDC/pDC Ratio, MDSC Frequency, and Th-17/Treg Ratio during Experimental Cerebral Malaria

**DOI:** 10.3390/pathogens13100877

**Published:** 2024-10-08

**Authors:** Saikat Mukherjee, Pronabesh Ghosh, Soubhik Ghosh, Anirban Sengupta, Samrat Sarkar, Rimbik Chatterjee, Atreyee Saha, Sriparna Bawali, Abhishek Choudhury, Altamas Hossain Daptary, Anwesha Gangopadhyay, Tarun Keswani, Arindam Bhattacharyya

**Affiliations:** 1 Immunology Laboratory, Department of Zoology, University of Calcutta, 35, Ballygunge Circular Road, Kolkata 700019, West Bengal, India; mukherjee.saikat053@gmail.com (S.M.); pronabesh2214@gmail.com (P.G.); soubhik1992@gmail.com (S.G.); ssarkar887@gmail.com (S.S.); rimbikrikchatterjee@gmail.com (R.C.); atreyeesaha43@gmail.com (A.S.); sriparnabawali0605@gmail.com (S.B.); abhishekrishi016@gmail.com (A.C.); hossainaltamas@gmail.com (A.H.D.); anweshagangopadhyay78937@gmail.com (A.G.); 2 Centre for Infectious Medicine, Department of Medicine Huddinge, Karolinksa Institutet, 14152 Stockholm, Sweden; online.asg@gmail.com; 3 Center for Immunological and Inflammatory Diseases, Department of Medicine, Massachusetts General Hospital, Harvard Medical School, Boston, MA 02129, USA

**Keywords:** malaria, MDSCs, Th17/Treg, mDC/pDC, rIL-33, immune balance

## Abstract

The onset of malaria causes the induction of various inflammatory markers in the host’s body, which in turn affect the body’s homeostasis and create several cerebral complications. Polarization of myeloid-derived suppressor cells (MDSCs) from the classically activated M1 to alternatively activated M2 phenotype increases the secretion of pro-inflammatory molecules. Treatment with recombinant IL-33 (rIL-33) not only alters this MDSC’s polarization but also targets the glycolysis pathway of the metabolism in MDSCs, rendering them less immunosuppressive. Along with that, the Helper T-cells subset 17 (Th17)/T regulatory cells (Tregs) ratio is skewed towards Th17, which increases inflammation by producing more IL-17. However, treating with rIL-33 also helps to restore this ratio, which brings back homeostasis. During malaria infection, there is an upregulation of IL-12 production from dendritic cells along with a distorted myeloid dendritic cells (mDC)/plasmacytoid dendritic cells (pDC) ratio towards mDCs promoting inflammation. Administering rIL-33 will also subvert this IL-12 production and increase the population of pDC in the host’s immune system during malaria infection, thus restoring mDC/pDC to homeostasis. Therefore, treatment with rIL-33 to reduce the pro-inflammatory signatures and maintenance of immune homeostasis along with the increase in survivability could be a potential therapeutic approach for cerebral malaria.

## 1. Introduction

There were 5 million more infected cases globally in 2022 than there were in 2021, totaling 249 million cases. The death rate decreased somewhat despite an increase in cases. A total of 608,000 people died from malaria in 2022 compared to 610,000 in 2021, with a large percentage of those deaths being African children, who are thought to carry approximately 90% of the global malaria burden [1,2]. While there has been a decrease in the number of deaths, it still remains one of the most common diseases in Africa and some parts of Asia [3]. Malaria is a mosquito-borne disease transmitted by the female *Anopheles* mosquito [4]. Protozoans belonging to the genus *Plasmodium* are the causative agents of malaria [5]. *Plasmodium falciparum* (*P. falciparum*), *Plasmodium vivax* (*P. vivax*), *Plasmodium malariae* (*P. malariae*), *Plasmodium knowlesi (P. knowlesi*), and *Plasmodium ovale* (*P. ovale*) are the five main pathogenic species to humans, among which, *P. falciparum* is responsible for the vast majority of mortality and is considered the most dangerous [6,7]. *P. falciparum* causes cerebral malaria (CM), and surviving patients show neurologic complications such as cognitive deficits, behavioral difficulties, motor alterations, and cortical blindness [8,9]. *Plasmodium berghei* ANKA (PbA) is a rodent-specific analog of *P. falciparum* causing experimental cerebral malaria (ECM), both with resemblances in terms of increased vascular leakage, upregulation of inflammatory cytokines, and many others [10,11]. Pathological conditions of CM or ECM depend on the balance between pro-inflammatory and regulatory immune responses and failing to maintain such homeostasis can lead to the development of cerebral complications [12,13]. Murine ECM shows very little intracerebral sequestration of infected red blood cells (iRBCs) but there is a strong pro-inflammatory cytokine response noted in the brain of the animal whereas human CM is documented with sequestration of iRBCs to the cerebral microvasculature together with inflammatory alterations [14]. The symptomatology and the immune homeostasis alterations are very similar in cases of ECM and CM and thus the ECM model has been extensively studied to understand this disease [15,16]. Blood–brain barrier breakdown and strong Th1 responses are associated with ECM, which promote inflammation [17]. The pro-inflammatory and counter-regulatory immune responses are controlled by the balance between T-helper cells subset 17 (Th17) and T-regulatory cells (Tregs) and during PbA infection, the Th17/Treg ratio skewed towards Th17 as the parasitic load increased, leading to an increase in serious pathological conditions and decrease in host survivability [18,19]. It has also been observed that in ECM, myeloid-derived suppressive cells (MDSCs) expand in an IL-6-dependent manner, further promoting disease pathogenesis [20]. MDSCs consist of a population of heterogeneous immature myeloid cells, which on differentiation give rise to granulocytes, macrophages, or dendritic cells (DCs); these cells have been reported to expand during tumor progression or chronic inflammation [21,22,23]. MDSCs are a population of cells possessing potent immune-suppressive roles, which are pathologically activated monocytes and neutrophils [24]. Usually, two subsets of MDSCs are delineated, termed granulocytic or polymorphonuclear (PMN-MDSCs), similar to neutrophils in terms of morphology, and monocytic (M-MDSCs), which are similar to monocytes [25]. MDSCs can be activated to two different phenotypic states, which are the classically activated M1 state promoting inflammation and alternatively, the activated M2 state with anti-inflammatory functions [26] and their immunosuppressive ability is metabolically majorly dependent on glycolysis and thus targeting their metabolic pathways is now being explored as a novel therapeutic measure in different diseases [27]. Also, dendritic cells (DCs) play a pivotal role during ECM, linking innate and adaptive immune responses at the host–pathogen interface. DCs function as a group of specialized cells functioning as antigen-presenting cells (APCs) and are classified into two subgroups, which are myeloid DCs (mDCs) and plasmacytoid DCs (pDCs), characterized by CD11c+CD11b+ and CD11c+CD45R/B220+ markers, respectively [28,29], and during PbA infection, it was noted that there is a skewed mDC/pDC towards mDC [28]. Thus, treatment with a Th2 or counter-regulatory cytokine can be one of the therapeutic approaches to reduce ECM complications. Interleukin-33 (IL-33), an IL-1 family nuclear cytokine [30], plays an important role in many inflammatory disorders, which include rheumatoid arthritis, allergic rhinitis, and others [26]. IL-33 mediates both innate and adaptive immune responses as it predominantly induces the production of Th2 cytokines, which include IL-4, IL-5, and IL-13 from the cells associated with innate immune signaling [31]. It has also been reported that IL-33 plays a pivotal role in controlling the expansion of regulatory T cells and effector functions, thus controlling immunosuppression and tissue repair [32]. IL-33 majorly targets the tissue-resident cells comprising mast cells, group 2 innate lymphoid cells (ILC2s), and Tregs [30]. Previous studies have reported the beneficial roles of IL-33 in clearing helminth infections [33]. Suppression of tumorigenicity 2 (ST2), a member of the IL-1 superfamily, is the receptor of IL-33. It exists in two isoforms, which include a soluble form (sST2) functioning as a decoy receptor and distrains free IL-33 while the other membrane-bound form is required for the proper functioning of IL-33, which mediates mast cell, Th2, Treg, and other immune responses via activation of the NF-κB signaling pathway [34]. Thus, in this study, we demonstrated the effects of rIL-33 not only on MDSCs’ expansion but also on their state of glucose metabolism, mDC/pDC ratio, in controlling inflammatory markers as well as how the Th17/Treg ratio is altered during the treatment with rIL-33 in experimental cerebral malaria.

## 2. Materials and Methods

### 2.1. Antibodies and Reagents

Anti-CD11b-PerCP-Cy5.5, anti-Gr-1-V450, anti-Gr-1-FITC, Ultra LEAF Purified anti-mouse Ly6G/Ly6C (Gr-1) with isotype control, anti-CD40-FITC, anti-CD80-PE, anti-CD86-PE, anti-CD25-FITC, anti-CD4-PerCP-Cy5.5, anti-IL-12-PE, anti-FOXP3-APC, anti-IL-17-APC fluorochrome-tagged mouse monoclonal antibodies (mAb) were purchased from Miltenyi Biotech (North Rhine-Westphalia, Germany)and BioLegend (San Diego, CA, USA). Other chemicals were purchased from Himedia (Mumbai, India) and MERCK Chemicals (Mumbai, India).

### 2.2. Ethical Statement and Induction of In Vivo Malaria Infection

Swiss Albino mice (each of 25–30 g weight, male, and aged 6–8 weeks) were housed in sterilized cages (three mice in each cage). Rodent chow was purchased from the National Institute of Nutrition, India, and filtered water was ad libitum to all cages. Experiments and animal handling were strictly according to the guidelines laid down by the ‘Committee for the Purpose of Control and Supervision of Experimental Animals (CPCSEA)’, Government of India (Registration No: 885/ac/05/CPCSEA). Institutional Animal Ethics Committee (IAEC), University of Calcutta (ZOO_AE_AB_2022_003), confirmed with the “Guide for the Care and Use of Laboratory Animals” as stated by the US National Institutes of Health. The parasitic strain of PbA was acquired from the National Institute of Malarial Research Centre, New Delhi. Red blood cells from parasitized mice (pRBC) were stored in liquid nitrogen. An amount of 1 × 10^6^ pRBC diluted in 100 µL PBS was injected in reservoir mice for amplification. Experimental mice were inoculated post-amplification with PbA (1 × 10^6^ pRBC diluted in 100 µL PBS) from reservoir mice by injecting them intraperitoneally (IP). Control mice were injected with an equivalent number of uninfected RBCs. The parasite load was calculated by the formula: Parasitemia (%) = [(number of infected erythrocytes)/(total number of erythrocytes counted) × 100]. Mice survival was also recorded daily [28].

### 2.3. Recombinant IL-33 Administration

From the initial stage of infection, mice were also treated with recombinant mouse Interleukin-33 (rIL-33) purchased from Gibco by intraperitoneal injection at a dose of 0.2 µg/mouse dissolved in 200 µL PBS daily from the day of infection (day 0). Mice were sacrificed 8 days post-infection when parasite load and mortality were recorded to be highest [20]. Uninfected mice used as experimental controls were also treated with rIL-33 for the first five days to study the drug effects.

### 2.4. MDSC Isolation from Spleen

MDSCs derived from mice spleens marked by CD11b+ Gr1+ population were isolated from uninfected control mice as well as PbA-infected mice and rIL-33-treated infected mice by using the EasySep™ Mouse MDSC (CD11b+ Gr1+) Isolation Kit purchased from StemCell Technologies (Vancouver, BC, Canada). This kit isolates MDSCs by the negative selection method from the single-cell suspensions of splenocytes. To maintain cell viability, 5% FBS in PBS solution was used during the whole process.

### 2.5. Determination of Arginase-1 Activity from Sorted MDSCs

Isolated MDSCs from uninfected, infected, and treated mice were used to determine NO secretion and Arg-1 activity. Arginase Activity Assay Kit (colorimetric) (ab180877) was used to determine Arg-1 activity. The 1 × 10^6^ cells were washed with cold PBS and then resuspended in 100 µL of ice-cold assay buffer and the cells were then homogenized. The homogenate was centrifuged, and the supernatant was collected to perform the assay by following the provided protocol.

### 2.6. Determination of Nitric Oxide (NO) Secretion from Sorted MDSCs by Griess Method

Sorted MDSCs from all experimental groups were cultured in RPMI-1640 culture media that was additionally supplemented with 10%FBS, 2 mM 1-glutamine; also, 1× antibiotic–antimycotic was provided in the culture media. The cell cultures were then maintained at an optimal temperature of 37 °C in a humidified atmosphere of 5% CO_2_ content. An amount of 1 µg/mL lipopolysaccharide solution was used to stimulate the cells for a day. Griess Reagent was then used to detect NO content in the culture supernatant [35].

### 2.7. Glycolysis Assay from Sorted MDSCs

Freshly sorted MDSCs from the single-cell suspension of spleens of mice from all experimental groups were used to study the glycolysis rate to assess their metabolic reprogramming. Glycolysis assay kit Abcam (Cambridge, United Kingdom) was used to measure the extracellular acidification rate (ECAR) due to lactate production during glycolysis by the fluorometric method at 380 nm excitation and 615 nm emission by using a microplate reader. The 2.5 × 10^5^ cells were harvested from spleens after 8 days post-infection and treatment cycle and were immediately used to take readings of the mean fluorescent readings corresponding to the RFU units continuously for 2 h at an interval of 12 min.

### 2.8. L-Lactate Assay from Sorted MDSCs

The cultured supernatant of sorted MDSCs was collected for lactic acid secretion analysis by using Abbkine CheKine™ Micro Lactate Assay Kit (Atlanta, GA, USA) and data were recorded by following the manufacturer’s protocols.

### 2.9. Flow Cytometric Analysis

The whole spleen was removed aseptically from euthanized mice of all experimental groups and single-cell suspensions were prepared by mechanical disruption using a cell strainer. The cell suspension was then treated with ACK lysis buffer to lyse all erythrocytes for five minutes and then washed with cold DMEM to stop the reaction. Splenocytes were then resuspended in FACS buffer (PBS containing 0.1% BSA and 0.04% EDTA-Na2) and incubated with fluorochrome-tagged antibodies and then incubated on ice for 30–40 mins for surface staining. Splenocytes were treated with phorbol 12-myristate 13-acetate (PMA, 10 nM, Sigma-Aldrich, St. Louis, MO, USA) and ionomycin (1 µM, EMD Biosciences Inc., Darmstadt, Germany) for five hours to stain for intracellular cytokines. For the last three hours of the culture, Brefeldin A was also added. Cells were fixed with 4% paraformaldehyde at room temperature and in dark conditions for about 30 min and then surface staining with fluorescent-tagged antibodies was performed followed by permeabilization using permeabilization buffer purchased from BioLegend and then stained with intracellular antibodies (anti-IL-12-PE, anti-FOXP3-APC, anti-IL-17-PE) at a temperature of 4 °C for 30 min. Data were then collected using BD FACSVerse and BD FACSAria III and analyzed using Flowjo software (v10) [18,20,28]. Gating strategies are provided in Figure S2.

### 2.10. Statistical Analysis

Data from different experimental groups were analyzed and statistical significance was determined using the Mann–Whitney *t*-test or Kruskal–Wallis test followed by Dunn’s multiple comparison post hoc test for nonparametric data and, for parametric data, one-way ANOVA followed by Tukey’s test or two-way ANOVA followed by Bonferroni post hoc analysis was performed. Graph Pad Prism (version 8.4.3) was used to calculate all the statistical significances. All the values are denoted as means ± standard deviations (SD) and *p* < 0.05 was taken to be significant.

## 3. Results

### 3.1. Treatment with rIL-33 Causes Restoration of Altered mDC/pDC Ratio

**Figure 1:**
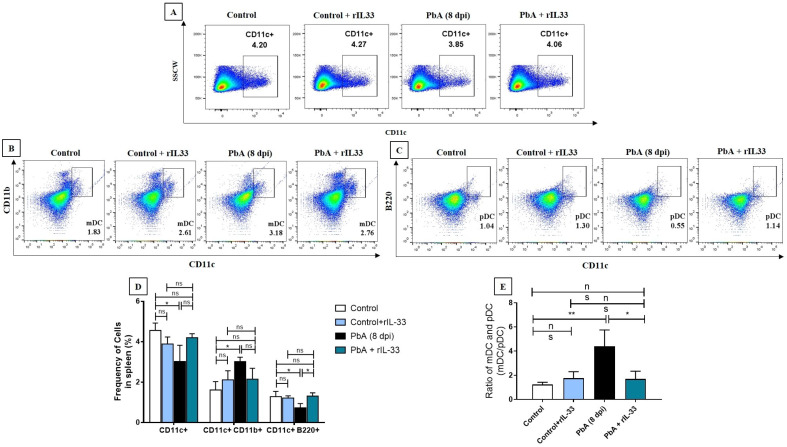
rIL-33 restores dendritic cell population as well as maintains mDC/pDC homeostasis. (**A**). Frequency of DCs (CD11c+) in all experimental groups; (**B**). Frequency of myeloid dendritic cells (CD11b+ CD11c+) with the splenocytes; (**C**). Frequency of plasmacytoid dendritic cells (pDCs) consisting of CD11c+B220+ cells within whole splenocytes in all experimental groups are shown; (**D**). The representative bar graphs of DC frequency as well the frequency of the subsets mDCs and pDCs; (**E**). Represents the ratio of mDC:pDC in all experimental groups. All experiments were performed at least three times independently and data in bar diagrams are represented as mean ± standard error of mean (SEM). Significance levels were tested using one-way ANOVA, where ns: non-significant, * *p* < 0.05, ** *p* < 0.01, *** *p* < 0.001.

Previously, the treatment with rIL-33 has been reported to reduce body weight loss and clinical scores related to ECM, and increase the survivability of mice [12], and a similar increase in the survivability of mice was recorded in our study (data given in Supplementary Figure S1). We hence decided to look for the beneficial roles of rIL-33 treatment on the immune system during malaria infection. Although the absolute number of CD11c+ dendritic cells increases in cases of ECM, their frequency decreases in the spleen [19]. rIL-33 treatment does not significantly change the frequency of DCs in the spleen (Figure 1A,D). DCs have broadly been classified into two subsets and they are mDCs and pDCs. These subsets have responses to different pathological conditions and evoke immune responses accordingly. During PbA infection, the prevalence of mDCs, which are CD11c+CD11b+, increases among the splenocytes whereas the prevalence of pDCs, which are CD11c+B220+, goes down, leading to the alteration in mDC/pDC, which is crucial in maintaining immune homeostasis, skewing it towards mDC. rIL-33 treatment does not affect the mDC population in infected mice (Figure 1B), but it increases the pDC population significantly (Figure 1C), thus restoring the mDC/pDC ratio to normalcy (Figure 1E).

### 3.2. Treatment with rIL-33 Causes Reduction in Inflammatory Marker Expression on DCs

**Figure 2:**
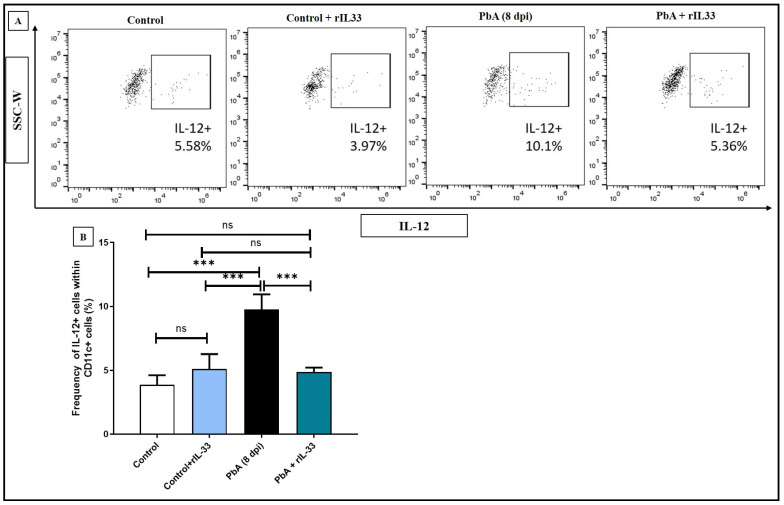
rIL-33 subverts the secretion of pro-inflammatory cytokine IL-12 from dendritic cells during ECM. (**A**). Represents dot plots of CD11c+ IL-12+ cells in whole splenocytes from all experimental groups. (**B**). Representative bar diagrams for CD11c+IL-12+ cells in whole splenocytes. The data in B are represented as mean ± SEM and one-way ANOVA was performed to test the significance levels, where ns: non-significant, * *p* < 0.05, ** *p* < 0.01, *** *p* < 0.001. All experiments were repeated independently at least three times.

**Figure 3:**
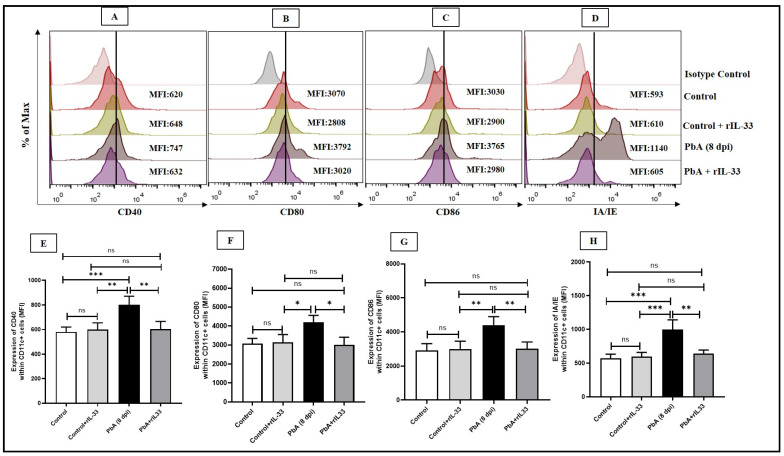
Expression levels of various co-stimulatory markers on CD11c+ dendritic cells are regulated by rIL-33. (**A**,**E**). Expression levels of CD40 within CD11c+ cells by MFI histograms and representative bar diagrams. (**B**,**F**). Expression levels of CD80 within CD11c+ cells by MFI histograms and representative bar diagrams. (**C**,**G**). Expression levels of CD86 within CD11c+ cells by MFI histograms and representative bar diagrams. (**D**,**H**). Expression levels of IA/IE (MHC II) within CD11c+ cells by MFI histograms and representative bar diagrams. The bar diagrams represent data as mean along with standard deviations. One-way ANOVA was performed to calculate the significance levels, where ns: non-significant, * *p* < 0.05, ** *p* < 0.01, *** *p* < 0.001. All experiments were repeated at least three times independently.

The levels of pro-inflammatory cytokine IL-12 were checked within the dendritic cells and it was noted that when rIL-33 treatment is given to PbA-infected mice, there is a significant decrease in the population of IL-12 secreting DCs, i.e., CD11c+IL12+ cell frequency decreases (Figure 2A,B). It was noted that co-stimulatory molecules like CD40, CD80, CD86, and MHCII showed an elevated expression in CD11c+ DCs in the spleens of infected mice. rIL-33 treatment dampened the expression of these molecules, thus providing a potential method to treat inflammation (Figure 3A–H).

### 3.3. rIL-33 Regulates the Expansion of MDSCs and Expression of Pro-Inflammatory Markers on Splenic MDSCs during ECM

**Figure 4:**
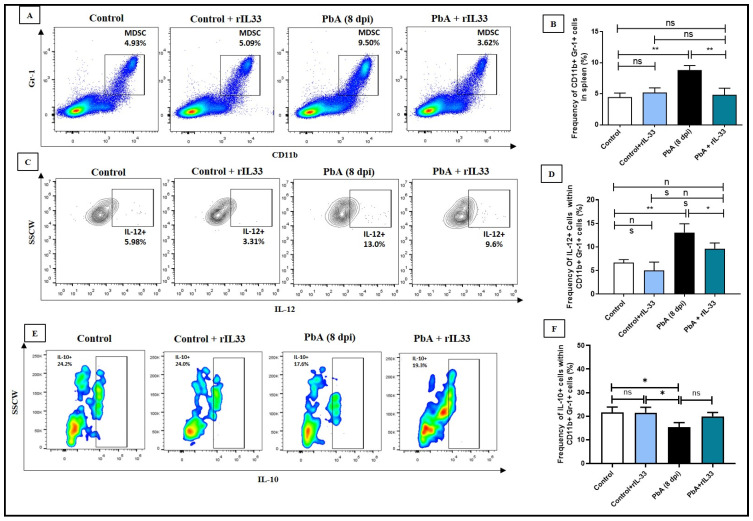
MDSC proliferation and secretion of pro-inflammatory and counter-regulatory cytokines are regulated by administration of rIL-33 during PbA infection. (**A**,**B**). Status of CD11b+ Gr-1+ cells (MDSCs) within splenocytes from all groups and the respective bar diagram. (**C**,**D**). Expression of IL-12 within CD11b+Gr1+ cells and the respective bar diagram. (**E**,**F**). Expression of IL-10 within CD11b+Gr1+ cells and the respective bar diagrams. All experiments were repeated a minimum of three times and ANOVA was performed to test the significance levels, where ns: non-significant, * *p* < 0.05, ** *p* < 0.01, *** *p* < 0.001. All data are represented as mean ± standard deviation (SD).

**Figure 5:**
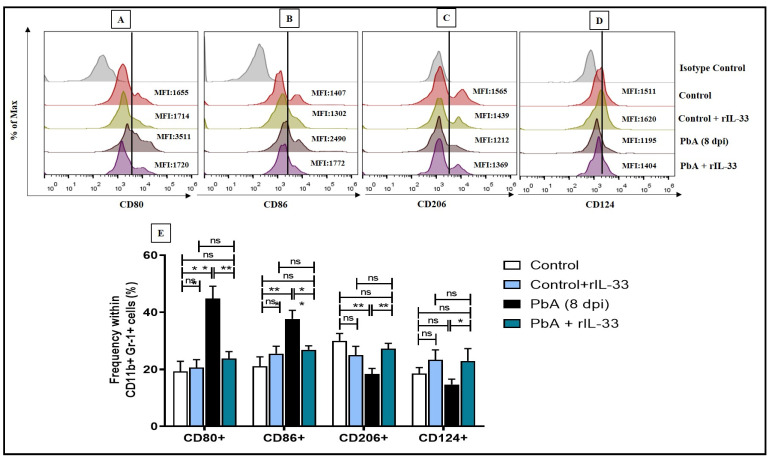
rIL-33 controls the M1/M2 polarization of MDSCs. The MFI plots are represented as (**A**). CD80; (**B**). CD86; (**C**). CD206; and (**D**). CD124. (**E**). Represents the representative bar diagram for the percentage changes in all co-stimulatory markers within CD11b+Gr1+ cells. All experiments were performed at least three times independently and one-way ANOVA was performed to test the significance levels. The bar diagram represents mean ± standard error of mean (SEM). (ns: non-significant, * *p* < 0.05, ** *p* < 0.01, *** *p* < 0.001).

Administration of rIL-33 treatment to malaria-infected mice has been shown to reduce the MDSC (CD11b+Gr1+) population in the spleen 8 days post-infection by flow cytometric analysis (Figure 4A,B). PbA infection causes the differential expression of various pro-inflammatory and counter-regulatory cytokines. Upon infection, there was an enhanced level of IL-12 expression noted in MDSCs whereas the IL-10 level decreased (Figure 4C–F). On rIL-33 treatment, the MDSC population, also positive for IL-12, was subverted; however, the IL-10+ MDSC population was not altered as such(Figure 4C–F). The expression of CD80 and CD86, which were found to be highly expressed on MDSCs during PbA infection, was found to decrease upon rIL-33 administration (Figure 5A,B,E). It was also noted that the expression of CD206 and CD124 increased when PbA-infected mice were treated with rIL-33 (Figure 5C–E). Thus, rIL-33 treatment caused the restoration of MDSC expansion along with other inflammatory markers in the spleens of PbA-infected mice at 8 dpi.

### 3.4. Treatment with rIL-33 Reduces the Suppressive Nature of MDSCs

**Figure 6:**
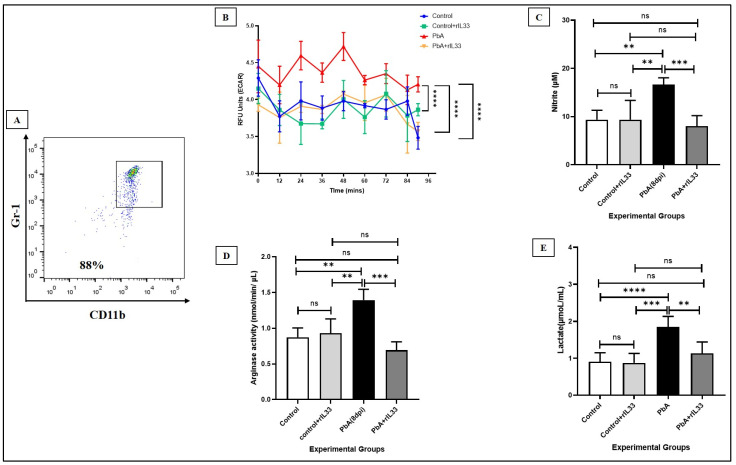
Metabolic reprogramming of MDSCs and their suppressibility are controlled by rIL-33 during PbA infection. (**A**). Characterization of MDSCs sorted from MACS by CD11b+Gr1+ double staining. (**B**). RFU units corresponding to the ECAR rates from live sorted MDSCs denoting the rate of glycolysis of these cells from all experimental groups. (**C**). Estimates of Nitric Oxide by Griess Assay from culture supernatants of sorted MDSCs. (**D**). Arginase activity from cell lysates of MDSCs from all experimental groups. (**E**). Estimates of lactate content from the cultured supernatants of sorted MDSCs. The bar diagrams represent means along with the standard deviations. All the experiments were repeated independently for a minimum of three times and one-way ANOVA was performed to test the significance levels. (ns: non-significant, * *p* < 0.05, ** *p* < 0.01, *** *p* < 0.001, **** *p* < 0.0001).

MDSCs are a group of suppressive cells, and it has been established that their suppressive effects towards CD4+ T cell proliferation are highest when the density of the respective cells is 1:1 in in vitro cultures [20]. The suppressive nature of MDSCs is due to the secretion of some characteristic molecules. Arginase-1 (Arg1) and inducible Nitric Oxide Synthase (iNOS) are reported to be the principal molecules secreted from MDSCs responsible for the suppressive nature [25,36]. We found that treatment with rIL-33 not only causes a marked decrease in the nitrite secretion in the culture supernatant of the sorted cells (Figure 6C) but also the arginase activity was found to be much lowered in the cell lysate of sorted MDSCs as compared to the matched infected groups (Figure 6D).

### 3.5. Metabolic Reprogramming of MDSCs during ECM

Reprogramming of the metabolism is a marked phenomenon of cancer wherein tumor cells undergo metabolic reprogramming to sustain their increasing energy demands to support their proliferation and survival. Similarly, MDSCs can also sense their environment and respond adequately by reprogramming their metabolism to the most efficient pathway to sustain the suppressive nature towards other immune cells [24] and glycolysis was affirmed as the main energy-driving pathway. During ECM, MDSCs showed a higher extracellular acidification rate as compared to cells from matched uninfected groups, which again reverted to normalcy upon treatment with rIL-33 (Figure 6B), which was also confirmed by the lactate secretion of these cells in their culture supernatant (Figure 6E). Thus, rIL-33 treatment further causes a shift in the metabolism from glycolysis, thus rendering them less immune-suppressive and restoring normal immune response to help clear the disease.

### 3.6. Treatment with rIL-33 Restores Distorted Th-17/Treg Balance

**Figure 7:**
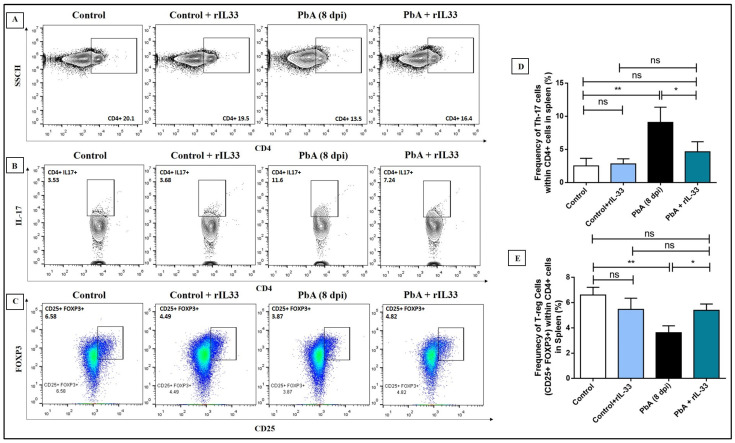
rIL-33 plays a pivotal role in maintaining Th17/Treg homeostasis during PbA infection. (**A**). Contour plots for CD4+ T-helper cells of all experimental groups. (**B**,**D**). CD4+IL-17+ (Th17) cells are represented by contour plots and the respective bar diagrams are shown. (**C**,**E**). CD4+CD25+FOXP3+ (Tregs) cells are represented by dot plots and the respective bar diagrams are shown. The bar diagrams represent means along with the standard error of mean. All the experiments were repeated independently for a minimum of three times and one-way ANOVA was performed to test the significance levels. (ns: non-significant, * *p* < 0.05, ** *p* < 0.01, *** *p* < 0.001).

Previous studies have reported Th17/Treg balance was important to maintain immune homeostasis [20,37]. CD4+IL-17+ (Th-17) cells were analyzed by flow cytometry at 8 dpi to understand the role of rIL-33 in inducing Th17. Significant reduction in the population of Th17 was observed in PbA-infected mice that were treated with rIL-33 than in those treated with PBS (Figure 7A,B,D). One of the major subsets of T-helper cells are T regulatory cells (Tregs), which have important counter-regulatory roles in infections and various other autoimmune diseases. During ECM, immune homeostasis maintained by the Th17/Treg ratio is skewed towards Th17 and a decrease in the Treg population was observed. Administration of rIL-33 caused the Treg population to increase, which led to the restoration of Th17/Treg balance (Figure 7C,E).

## 4. Discussion

The largest secondary lymphoid organ in the body is the spleen, which functions as a massive blood filter, removing anomalous or senescent red blood cells (RBCs) and pathogenic organisms from circulation [38,39,40]; it also hosts various immunological functions both in human and rodent species [41]. Malaria infection is the most prevalent cause of spleen rupture and splenomegaly is a landmark symptom of the disease [42]. This organ functions in the clearance of parasitized RBCs (pRBCs) and also in activating other immune cells by acting as a major antigen-presenting site during disease pathogenesis [29,43]. DCs play a pivotal role in mounting immune responses by bridging the innate immune system’s function of detecting microbial particles to the adaptive immune system in targeting them [44]. In this study, we noted that although there were no significant changes in the frequency of DCs in the spleen during malaria infection, there was an increase in the mDC/pDC ratio, leading to disease pathogenesis, which was compensated for by treatment with rIL-33. IL-12 cytokine secretion is found to be important in mounting pro-inflammatory immune responses during various infections [38] and is also increased during ECM [20]. DCs have been classified broadly into two subsets, which are mDC and pDC, which have different phenotypes and functions both in mice and humans. Among the subsets, mDCs respond to bacteria and other similar pathogens and can secrete more IL-12 to evoke Th1 responses while pDCs act against viruses with a high interferon-alpha production to evoke both Th1 and Th2 responses [45]. Our results show an increased mDC population upon PbA infection and a concomitant increase in the IL-12-secreting CD11c+ cells, which might be due to the mDCs only, which are CD11c+ CD11b+ cells, and treatment with rIL-33 led to a decrease in the DC population secreting IL-12. Dendritic cell costimulatory markers are essential for both initiating and controlling the immune response. Certain markers, such as CD80, CD86, and CD40, give T lymphocytes the secondary signals they need when antigen is presented. Through this interaction, a strong immune response is produced by ensuring that T cells are completely activated and capable of responding to pathogens. An effective immune response may be thwarted by T cells that become anergic or tolerant in the absence of these costimulatory signals [46]. Expression analysis of other co-stimulatory molecules like CD80, CD86, CD40, and IA/IE was found to be regulated by rIL-33. The expression of all these markers increased upon infection and administration of rIL-33 subverts them. MDSCs play a pivotal role in modulating immune responses in cancer, chronic infectious disease, and other auto-immune and pathological conditions [47], and were also found to play an important role in ECM [20] and are also regulated by IL-33. On administration of rIL-33, not only MDSC expansion but also the expression of pro-inflammatory and counter-regulatory markers like CD80, CD86, CD206, and CD124 was altered. Meanwhile, in infectious conditions, the expression of pro-inflammatory markers CD80 and CD86 was found to be elevated and the expression of counter-regulatory markers CD206 and CD124 was reduced, causing the polarization of MDSCs to a classically activated M1 phenotype. rIL-33 treatment reversed this scenario and led the MDSCs to a more alternately activated M2 phenotypic state. Apart from the phenotypic state, rIL-33 was also found to regulate their metabolic state. Glycolysis, which was related to its increased immune-suppressive function, was found to be subverted upon administration of rIL-33 during ECM. Also, it was noted that Th17 cells characterized by the secretion of IL17, a strong inflammatory cytokine [48], and Treg cells, another subset of T-helper cells involved in immune responses [49], are affected by the treatment with rIL-33. During malaria infection, the Th17/Treg homeostatic imbalance was also found to be restored on administration of rIL-33 by increasing Treg cell populations during infections.

## 5. Conclusions

**Figure 8:**
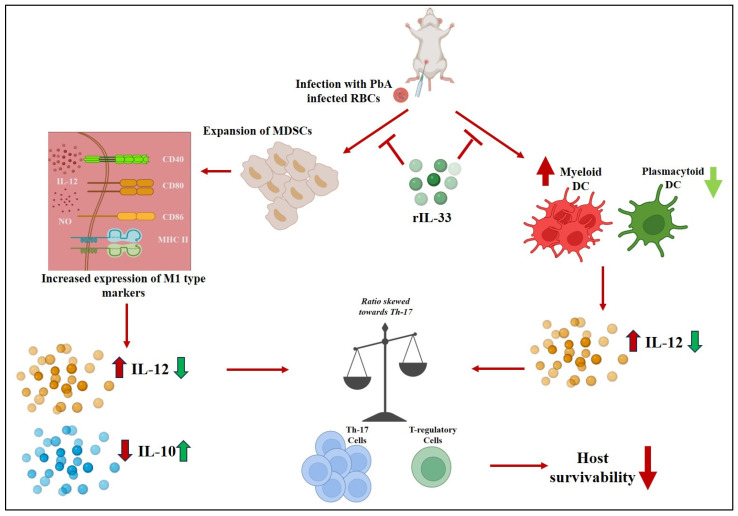
A schematic representation of IL-33 mode of action. IL-33 causes in vitro polarized TH2 cells to produce TH2-associated cytokines and activates MAP kinases and NF-kappaB. In vivo, IL-33 causes mucosal organs to undergo significant pathological alterations and stimulates the expression of IL-4, IL-5, and IL-13. IL-33 has also been found to be beneficial in helminth parasite clearance and similar trends were found in cases of malarial infection with PbA. Upon infection with parasitized RBCs (pRBCs), splenomegaly was observed along with the expansion of MDSCs and the expression of M-1 type markers on them and these cells secrete a high amount of pro-inflammatory cytokines (IL-12) with a reduced secretion of counter-regulatory cytokine (IL-10). Also, the mDCs expand and the mDC/pDC ratio becomes skewed towards mDCs and there is an increased production of IL-12 as well. All these imbalances lead to an increase in Th-17 cells, ultimately leading to compromised host survivability. Upon induction of IL-33, these immunological imbalances are reverted, leading to parasite clearance, reduced disease load, and an increase in host survivability.

Malaria infection is marked mainly by inflammation of the spleen along with an increase in various inflammatory markers and T cell subsets that further promote inflammatory responses. Previous studies have reported that the Th17/Treg ratio and mDC/pDC ratio were crucial in determining the disease pathogenesis, and rIL-33 was found to regulate these cells effectively. Through the IL-1 receptor ST2, IL-33 is known to mediate its biological activities by activating MAP kinases and NF-kappaB. It was also found to stimulate the production of TH2-associated cytokines from TH2-polarized cells in vitro. In vivo experiments have revealed that IL-33 causes significant pathogenic alterations in the mucosal organs by inducing the expression of IL-4, IL-5, and IL-13 [50]. Among various other cells promoting disease severity, MDSCs play a pivotal role, and they reprogram their metabolism to further promote their suppressive nature. rIL-33 played a pivotal role in modulating the suppressive nature of MDSCs as well as controlling their metabolic state, rendering them less proliferating. Also, the administration of rIL-33 controlled the secretion of various pro-inflammatory and anti-inflammatory cytokines’ secretion through DCs and MDSCs but reduced the secretion of pro-inflammatory counterparts. The mode of action of rIL-33 during PbA infection is represented in Figure 8, which summarizes all our findings and states the beneficial role of rIL-33 during malaria infection. Hence, we may conclude that rIL-33 treatment was found to resolve inflammation, control the metabolism of MDSCs, and provide better disease-control outcomes.

## Data Availability

Data will be made available on request.

## Supplementary Materials

The following supporting information can be downloaded at: https://www.mdpi.com/article/10.3390/pathogens13100877/s1, Figure S1: The Kaplan-Meyer Survivability curve shows increased survival rate in mice treated that were infected with PbA and treated with rIL-33 as compared to PbA infected mice treated with PBS. The PbA mice group showed 90% mortality with 8th and 100% mortality within 13th day where as significant increase in survivability is shown in PbA+rIL33 group. Figure S2: Gating strategies of MDSCs, pDC and mDCs; Table S1: List of antibodies used in flowcytometry experiments with their sources mentioned.; Table S2: Panels for combination of antibodies in multiple staining for flow cytometry; Table S3: List of Reagents and Materials used for various experiments [51].

## References

[1] World Health Organizatio (2023). World Malaria Report 2023.

[2] World Health Organization (2022). World Malaria Report 2022.

[3] Talapko J., Škrlec I., Alebić T., Jukić M., Včev A. (2019). Malaria: The past and the present. Microorganisms.

[4] Ripathi A.K., Mlambo G., Kanatani S., Sinnis P., Dimopoulos G. (2020). Plasmodium falciparum Gametocyte Culture and Mosquito Infection Through Artificial Membrane Feeding. J. Vis. Exp..

[5] Sato S. (2021). Correction to: Plasmodium—A brief introduction to the parasites causing human malaria and their basic biology. J. Physiol. Anthropol..

[6] Luzolo A.L., Ngoyi D.M. (2019). Cerebral malaria. Brain Res. Bull..

[7] Amir A., Cheong F.W., de Silva J.R., Liew J.W.K., Lau Y.L. (2018). Plasmodium knowlesi malaria: Current research perspectives. Infect. Drug Resist..

[8] Leão L., Puty B., Dolabela M.F., Povoa M.M., Gecy Y., Né D.S., Eiró L.G., Carolina N., Fagundes F., Maia L.C. (2020). Association of cerebral malaria and TNF- α levels: A systematic review. BMC Infect. Dis..

[9] Rosa-Gonçalves P., Ribeiro-Gomes F.L., Daniel-Ribeiro C.T. (2022). Malaria Related Neurocognitive Deficits and Behavioral Alterations. Front. Cell. Infect. Microbiol..

[10] Movila A., Sohet F., Girgis N.M., Gundra U.M. (2014). Experimental Cerebral Malaria Pathogenesis—Hemodynamics at the Blood Brain Barrier. PLoS Pathog..

[11] White N.J., Turner G.D.H., Medana I.M., Dondorp A.M., Day N.P.J. (2009). The murine cerebral malaria phenomenon. Trends Parasitol..

[12] Besnard A., Guabiraba R., Niedbala W., Palomo J. (2015). IL-33-Mediated Protection against Experimental Cerebral Malaria Is Linked to Induction of Type 2 Innate Lymphoid Cells, M2 Macrophages and Regulatory T Cells. PLoS Pathog..

[13] Mabbott N.A. (2018). The Influence of Parasite Infections on Host Immunity to Co-infection with Other Pathogens. Front. Immunol..

[14] Nishanth G., Schlüter D. (2019). Blood–Brain Barrier in Cerebral Malaria: Pathogenesis and Therapeutic Intervention. Trends Parasitol..

[15] Cimperman C.K., Pena M., Gokcek S.M., Theall B.P., Patel M.V., Sharma A., Qi C.F., Sturdevant D., Miller L.H., Collins P.L. (2023). Cerebral Malaria Is Regulated by Host-Mediated Changes in Plasmodium Gene Expression. MBio.

[16] Huang B., Pearman E., Kim C. (2015). Mouse Models of Uncomplicated and Fatal Malaria. Bio-Protocol.

[17] Guo J., Waknine-grinberg J.H., Mitchell A.J., Barenholz Y., Golenser J. (2014). Reduction of Experimental Cerebral Malaria and Its Related Proinflammatory Responses by the Novel Liposome-Based β-Methasone Nanodrug. BioMed Res. Int..

[18] Keswani T., Bhattacharyya A. (2014). Differential role of T regulatory and Th17 in Swiss mice infected with Plasmodium berghei ANKA and Plasmodium yoelii. Exp. Parasitol..

[19] Keswani T., Sarkar S., Sengupta A., Bhattacharyya A. (2016). Role of TGF-β and IL-6 in dendritic cells, Treg and Th17 mediated immune response during experimental cerebral malaria. Cytokine.

[20] Mukherjee S., Ghosh S., Sengupta A., Sarkar S., Keswani T., Chatterjee R., Bhattacharyya A. (2022). IL-6 dependent expansion of inflammatory MDSCs (CD11b+ Gr-1+) promote Th-17 mediated immune response during experimental cerebral malaria. Cytokine.

[21] Umansky V., Blattner C., Gebhardt C., Utikal J. (2016). The role of myeloid-derived suppressor cells (MDSC) in cancer progression. Vaccines.

[22] Gabrilovich D.I., Ostrand-Rosenberg S., Bronte V. (2014). Coordinated regulation of myeloid cells by tumours. Bone.

[23] Dysthe M., Parihar R. (2020). Myeloid-Derived Suppressor Cells in the Tumor Microenvironment. Adv. Exp. Med. Biol..

[24] Veglia F., Sanseviero E., Gabrilovich D.I. (2021). Myeloid-derived suppressor cells in the era of increasing myeloid cell diversity. Nat. Rev. Immunol..

[25] Gabrilovich D.I. (2017). Myeloid-derived suppressor cells. Cancer Immunol. Res..

[26] Liew F.Y. (2012). IL-33: A Janus cytokine. Ann. Rheum. Dis..

[27] Li X., Li Y., Yu Q., Qian P., Huang H., Lin Y. (2021). Metabolic reprogramming of myeloid-derived suppressor cells: An innovative approach confronting challenges. J. Leukoc. Biol..

[28] Keswani T., Sengupta A., Sarkar S., Bhattacharyya A. (2015). Dendritic cells subsets mediated immune response during Plasmodium berghei ANKA and Plasmodium yoelii infection. Cytokine.

[29] Banchereau J., Briere F., Caux C., Davoust J., Lebecque S., Liu Y., Pulendran B., Palucka K. (2000). Mmunobiology of. Annu. Rev. Immunol..

[30] Cayrol C., Girard J.P. (2018). Interleukin-33 (IL-33): A nuclear cytokine from the IL-1 family. Immunol. Rev..

[31] Pinto S.M., Subbannayya Y., Rex D.A.B., Raju R., Chatterjee O., Advani J., Radhakrishnan A., Keshava Prasad T.S., Wani M.R., Pandey A. (2018). A network map of IL-33 signaling pathway. J. Cell Commun. Signal..

[32] Braun H., Afonina I.S., Mueller C., Beyaert R. (2018). Dichotomous function of IL-33 in health and disease: From biology to clinical implications. Biochem. Pharmacol..

[33] Moro K., Yamada T., Tanabe M., Takeuchi T., Ikawa T., Kawamoto H., Furusawa J.I., Ohtani M., Fujii H., Koyasu S. (2010). Innate production of TH 2 cytokines by adipose tissue-associated c-Kit+ Sca-1+ lymphoid cells. Nature.

[34] Griesenauer B., Paczesny S. (2017). The ST2/IL-33 axis in immune cells during inflammatory diseases. Front. Immunol..

[35] Ghosh P., Mukherjee S., Ghosh S., Gangopadhyay A., Keswani T., Sengupta A., Sarkar S., Bhattacharyya A. (2023). Estimating nitric oxide (NO) from MDSCs by Griess method. Methods Cell Biol..

[36] Hegde S., Leader A.M., Merad M. (2021). MDSC: Markers, development, states, and unaddressed complexity. Immunity.

[37] Ghosh S., Mukherjee S., Sengupta A., Chowdhury S., Sarkar S., Keswani T., Bhattacharyya A. (2022). CD4+IL9+ (Th9) cells as the major source of IL-9, potentially modulate Th17/Treg mediated host immune response during experimental cerebral malaria. Mol. Immunol..

[38] Lewis S.M., Williams A., Eisenbarth S.C. (2019). Structure and function of the immune system in the spleen. Sci Immunol..

[39] Aliyu M., Zohora F., Akbar Saboor-Yaraghi A. (2021). Spleen in innate and adaptive immunity regulation. AIMS Allergy Immunol..

[40] Krücken J., Mehnert L.I., Dkhil M.A., El-Khadragy M., Benten W.P.M., Mossmann H., Wunderlich F. (2005). Massive destruction of malaria-parasitized red blood cells despite spleen closure. Infect. Immun..

[41] Faucher J.F., Créantor C., Hustache-Mathieu L., Chirouze C., Millon L., Hoen B. (2006). Paludisme à Plasmodium falciparum d’évolution atypique chez un patient splénectomisé [Atypical course of falciparum malaria in an asplenic patient]. Presse Med..

[42] del Portillo H.A., Ferrer M., Brugat T., Martin-Jaular L., Langhorne J., Lacerda M.V.G. (2012). The role of the spleen in malaria. Cell. Microbiol..

[43] Ghosh D., Stumhofer J.S. (2021). The spleen: “epicenter” in malaria infection and immunity. J. Leukoc. Biol..

[44] Mellman I. (2013). Dendritic cells: Master regulators of the immune response. Cancer Immunol. Res..

[45] Nizzoli G., Krietsch J., Weick A., Steinfelder S., Facciotti F., Gruarin P., Bianco A., Steckel B., Moro M., Crosti M. (2013). Human CD1c+ dendritic cells secrete high levels of IL-12 and potently prime cytotoxic T-cell responses. Blood.

[46] Hubo M., Trinschek B., Kryczanowsky F., Tuettenberg A., Steinbrink K., Jonuleit H. (2013). Costimulatory molecules on immunogenic versus tolerogenic human dendritic cells. Front. Immunol..

[47] Youn J.I., Gabrilovich D.I. (2010). The biology of myeloid-derived suppressor cells: The blessing and the curse of morphological and functional heterogeneity. Eur. J. Immunol..

[48] Tesmer L.A., Lundy S.K., Sarkar S., Fox D.A. (2008). Th17 cells in human disease. Immunol. Rev..

[49] Rocamora-Reverte L., Melzer F.L., Würzner R., Weinberger B. (2021). The Complex Role of Regulatory T Cells in Immunity and Aging. Front. Immunol..

[50] Schmitz J., Owyang A., Oldham E., Song Y., Murphy E., McClanahan T.K., Zurawski G., Moshrefi M., Qin J., Li X. (2005). IL-33, an interleukin-1-like cytokine that signals via the IL-1 receptor-related protein ST2 and induces T helper type 2-associated cytokines. Immunity.

[51] Brown W.E., Hu J.C., Athanasiou K.A. (2016). Ammonium-Chloride-Potassium Lysing Buffer Treatment of Fully Differentiated Cells Increases Cell Purity and Resulting Neotissue Functional Properties. Tissue Eng. Part C Methods.

