# Supplementary material for: Administration of rIL-33 Restores Altered mDC/pDC Ratio, MDSC Frequency, and Th-17/Treg Ratio during Experimental Cerebral Malaria
Source: Pathogens. 2024 Oct 8;13(10):877. doi: 10.3390/pathogens13100877 (PMC11509898; doi:10.3390/pathogens13100877)

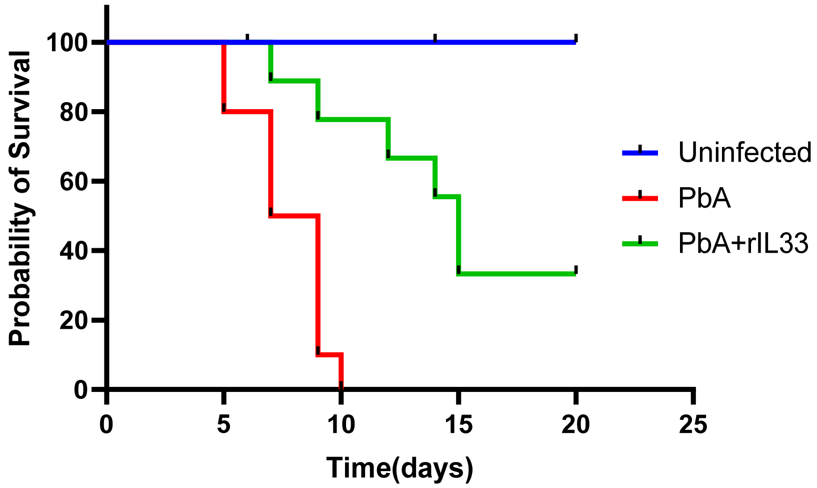

Figure S1: The Kaplan-Meyer Survivability curve shows increased survival rate in mice treated that were infected with PbA and treated with rIL-33 as compared to PbA infected mice treated with PBS. The PbA mice group showed 90% mortality with 8th and 100% mortality within 13th day where as significant increase in survivability is shown in PbA+rIL33 group.

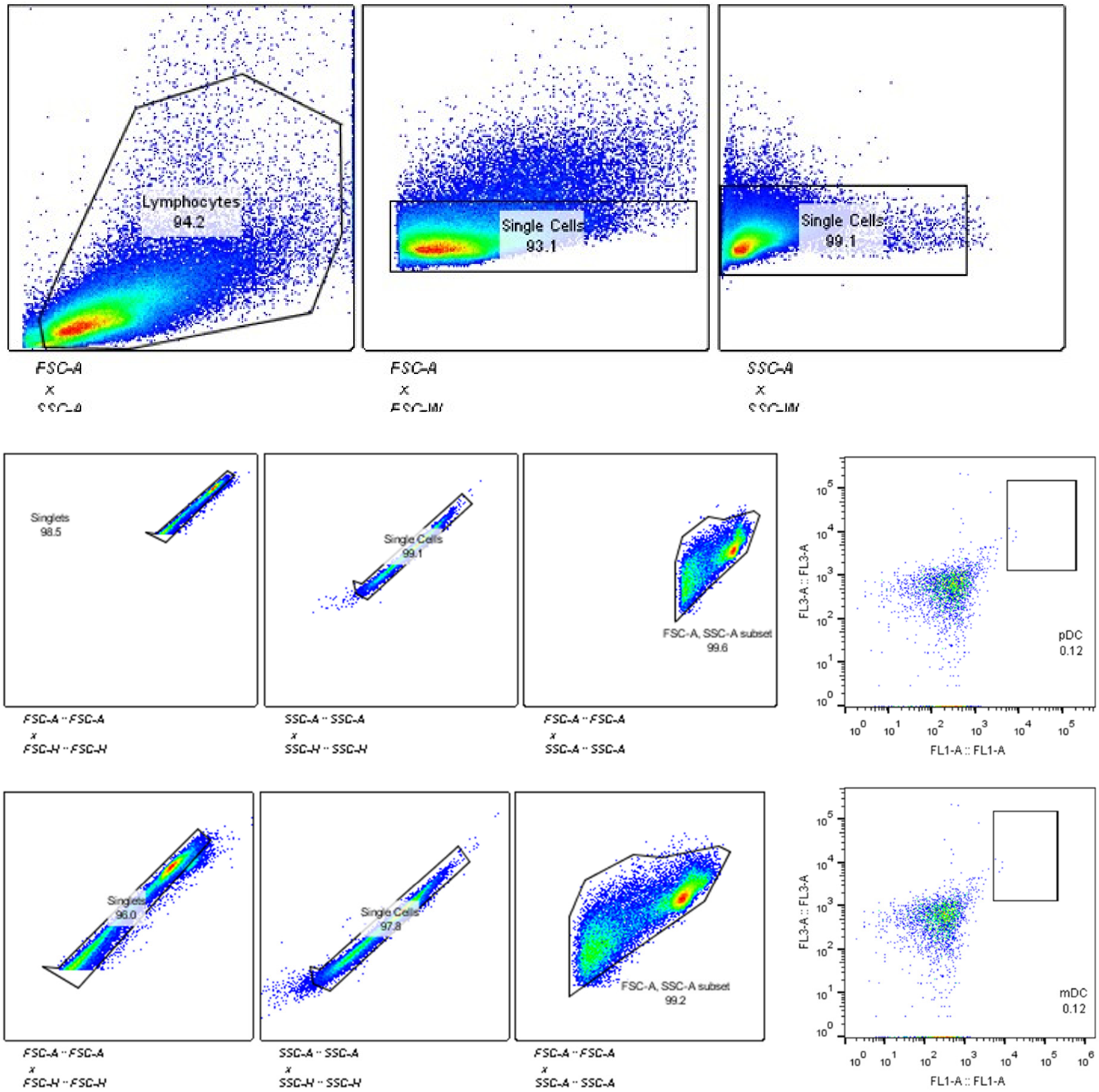

Figure S2: Gating strategies of MDSCs , pDC and mDCs.

Serial Number	Name of Antibody	Fluorescent tag	Name of company	Lot No.
1.	CD11b	PerCP-Cy5.5	BioLegend	B283049
2.	F4/80	APC	Miltenyi Biotech	5170608220
3.	Gr-1	V450	BD-Horizon	2223884
4.	Gr-1	FITC	BioLegend	B271251
5.	MDSC Flow cocktail 2		BioLegend	B260782
6.	CD11c	FITC	BD Pharmigen	61713
7.	B220	PerCP	Miltenyi Biotech	5170608224
8.	CD40	FITC	BD-Pharmigen	20391
9.	CD80	PE	BioLegend	B181610
10.	CD86	PE	BioLegend	B136226
11.	CD124	PE	BioLegend	B245608
12.	IA/IE	FITC	BioLegend	B179313
13.	CD4	PercpCy5.5	BioLegend	B276680
14.	IL-17A	APC	Miltenyi Biotech	5190201304
15.	RORγT	APC	BioLegend	5190116029
16.	pSTAT3 (Tyr705)	Alexa Fluor 488	BioLegend	B229258
17.	FOXP3	APC	Miltenyi Biotech	5190116439
18.	CD25	FITC	Miltenyi Biotech	5190110375
19.	IL-12	PE	BD Pharmigen	20152

Table S1: List of antibodies used in flowcytometry experiments with their sources mentioned.

Panel	V450	FITC	PE	PERCP Cy5.5	APC
1		CD11c	IL-12	B220	CD8
2	GR-1	CD11c	IL-12	CD11b	CD8
3	GR-1	CD40	CD80	CD11b	CD11c
4		GR-1	CD86	CD11b	CD11c
5	GR-1	IA/IE	CD206	CD11b	CD11c
6		GR-1	CD124	CD11b	
7		CD25	IL-17	CD4	FOXP3

Table S2: Panels for combination of antibodies in multiple staining for flow cytometry

**Names of Reagent/ Materials**	**Manufacturer**	**Catalog No.**
Phosphate Buffered Saline	HIMEDIA	M1452
Cell Strainer	HIMEDIA	TCP025-1X50NO
Fetal Bovine Serum	GIBCO	11550356
Recombinant Mouse IL-33	GIBCO	PMC4044
RPMI Medium 1640	GIBCO	31800-022
Antibiotic Antimycotic Solution	HIMEDIA	A002A-20ML
L-Glutamine 200mM	HIMEDIA	TCL012-20ML
L-Lactic Acid/Lactate (LA) Colorimetric Assay Kit 96 WELLS	ABBKINE	ABK1012Q09
Glycolysis AssayKit (Extracellular Acidificatiion)	ABCAM	AB197244
Lipopolysaccharides from E.coli	SIGMA-ALDRICH	L5543
Intracellular Staining Permeabilization Wash Buffer (10X)	BioLegend	421002

Table S3: List of Reagents and Materials used for various experiments.

ACK buffer treatment

The composition of the ACK buffer was 10 mM potassium bicarbonate, 97.3 µM EDTA tetrasodium salt, and 154.4 mM ammonium chloride. This is equivalent to 1. L of ultrapure water containing 8.26 g of ammonium chloride, 1.0 g of potassium bicarbonate, and 0.037 g of EDTA. Before used, this solution needs to be sterile filtered.

Similar compositions have been used in other studies like

1. Brown WE, Hu JC, Athanasiou KA. Ammonium-Chloride-Potassium Lysing Buffer Treatment of Fully Differentiated Cells Increases Cell Purity and Resulting Neotissue Functional Properties. Tissue Eng Part C Methods. 2016 Sep;22(9):895-903. doi: 10.1089/ten.TEC.2016.0184. PMID: 27553086; PMCID: PMC5035916.

Violin Plots of the data Sets

a. Expression of IL12 within CD11c+ Dendritic cells

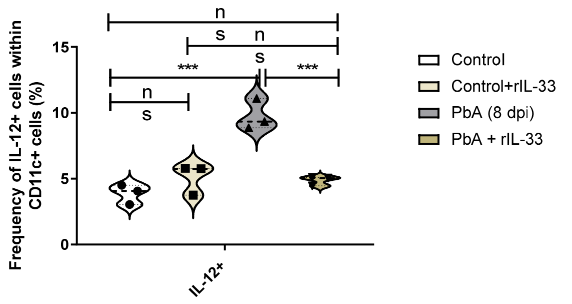

b. Expression of various co-stimulatory markers within CD11c+ Dendritic cells

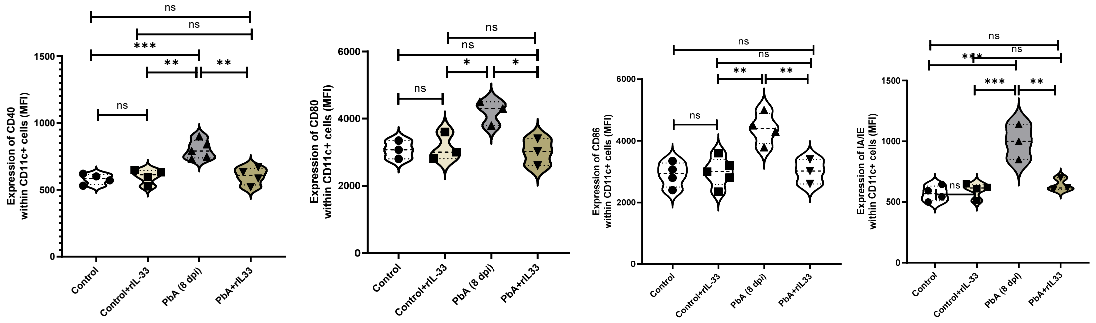

c. Frequency of MDSCs (CD11b+Gr1+) and the various co-stimulatory markers within them

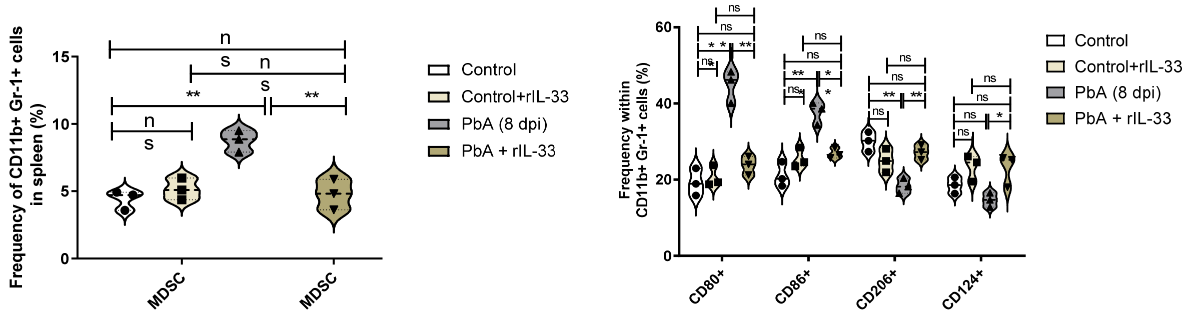

d. Expression of Cytokines (IL-12 and IL-10) within MDSCs

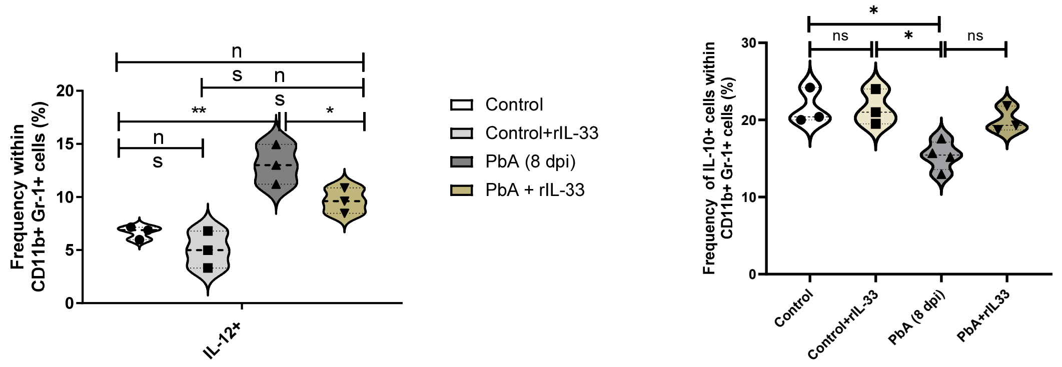

Supplement: Supplementary file 1 [file pathogens-13-00877-s001.zip › pathogens-3096259-supplementary.pdf]
